# Supplementary material for: Machine learning applied to enzyme turnover numbers reveals protein structural correlates and improves metabolic models
Source: Nat Commun. 2018 Dec 7;9:5252. doi: 10.1038/s41467-018-07652-6 (PMC6286351; doi:10.1038/s41467-018-07652-6)
Supplement: Supplementary file 1 — Supplementary Information [file 41467_2018_7652_MOESM1_ESM.pdf]

Supplementary Information

Machine learning applied to enzyme turnover numbers reveals protein structural correlates and improves metabolic models

Heckmann et al.

Contents

Supplementary Figures 2
Supplementary Tables 10
Supplementary References 12

Supplementary Figures

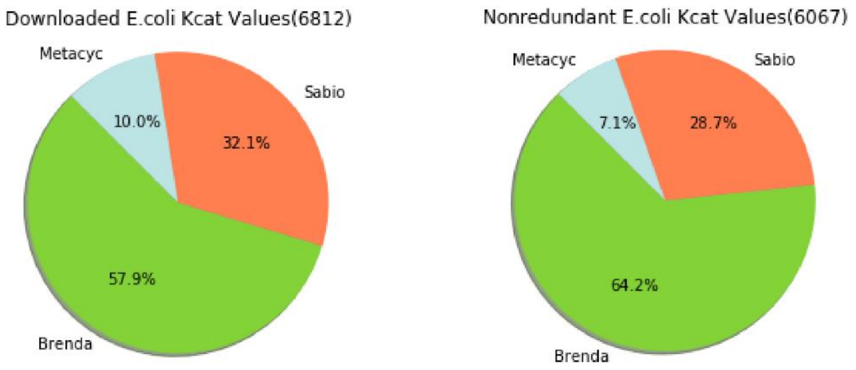

Supplementary Figure 1: Data contribution to *in vitro* k_{cat} values before and after the removal of redundant data points. Removal is done at two levels: within the same database and in between databases. Within the same database, repeated k_{cat} values refer to the same reaction done in the same paper are deleted. In between databases, preferences to the BRENDA and then the Metacyc database are given during deletion.

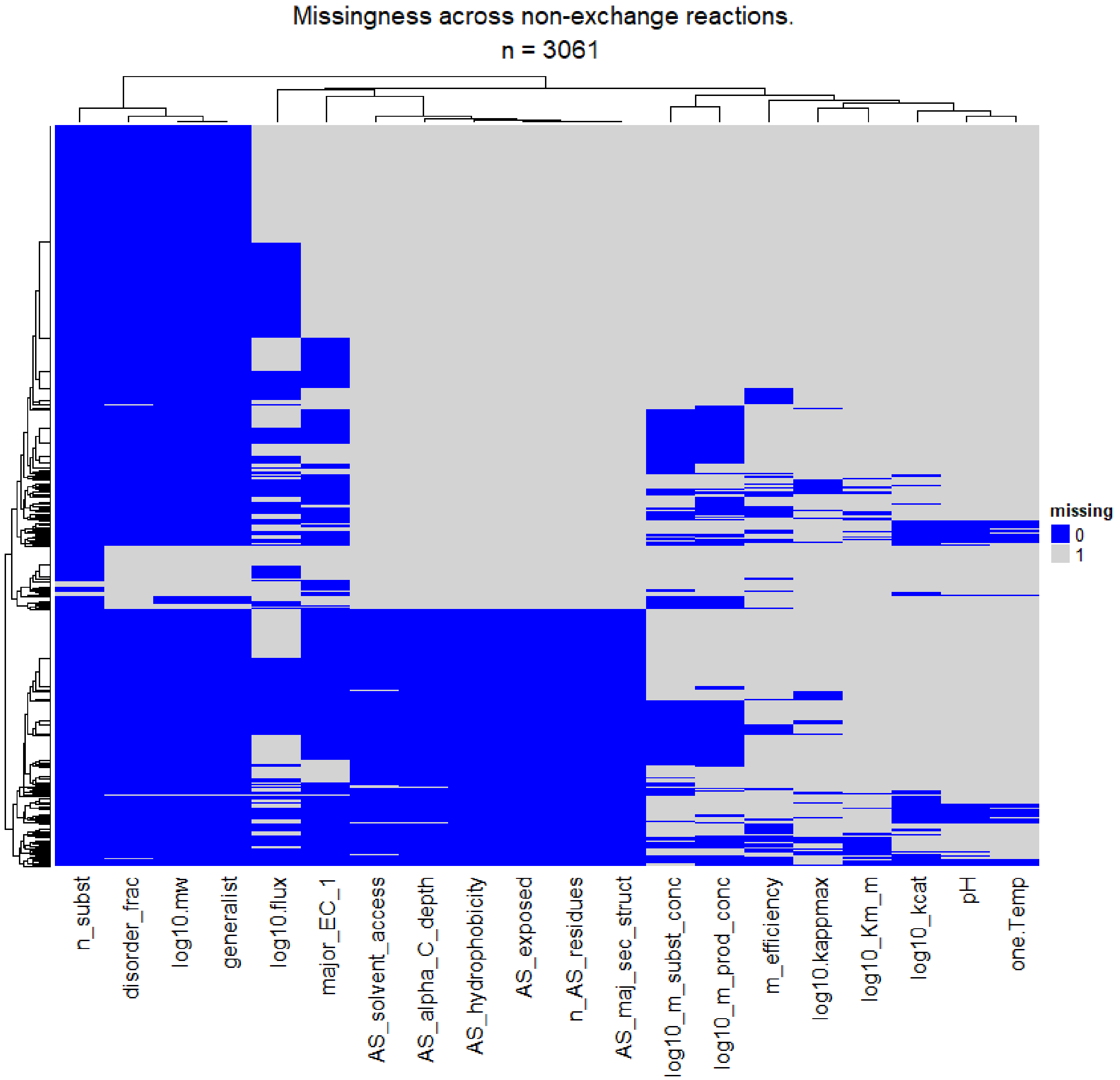

Supplementary Figure 2: Missing input and output values in the modelling process. Rows and columns are were clustered using complete linkage. Grey fields indicate missing data.

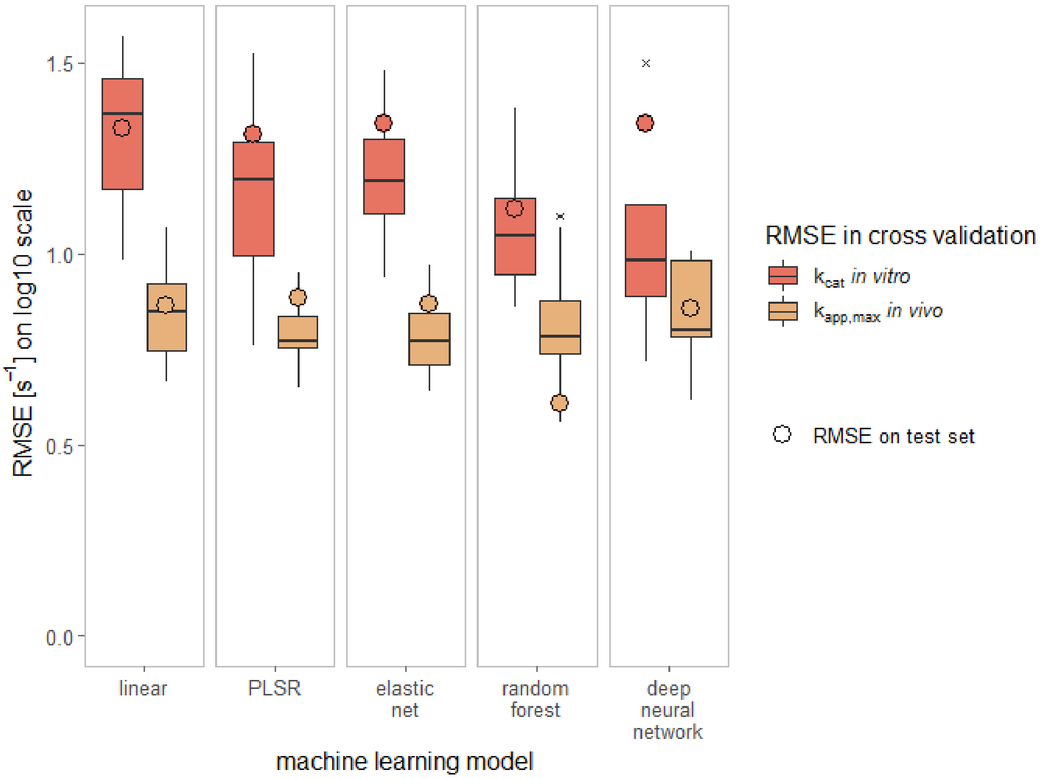

Supplementary Figure 3: Cross-validated machine learning model performances for $k_{app,max}$ and k_{cat} *in vitro* as measured by root mean squared error (RMSE). Related to Figure 2. Center lines show the median RMSE across 5 times repeated 5-fold cross-validation, except for the deep learning case, where a single iteration of 5-fold cross-validation is shown. Box limits represent the 1st and 3rd quartiles, whiskers extend to values that lie within the 1.5x interquartile range, and the remaining points are shown as outliers (marked x). Circles show RMSE for a test set consisting of 20% of the available samples that were not used for hyperparameter optimization. This resulted in a training set of 172 observations of k_{cat} *in vitro* and 106 observations of $k_{app,max}$. For the test set, 43 and 27 observations were used for k_{cat} *in vitro* and $k_{app,max}$, respectively. See Methods for details on hyperparameter optimization.

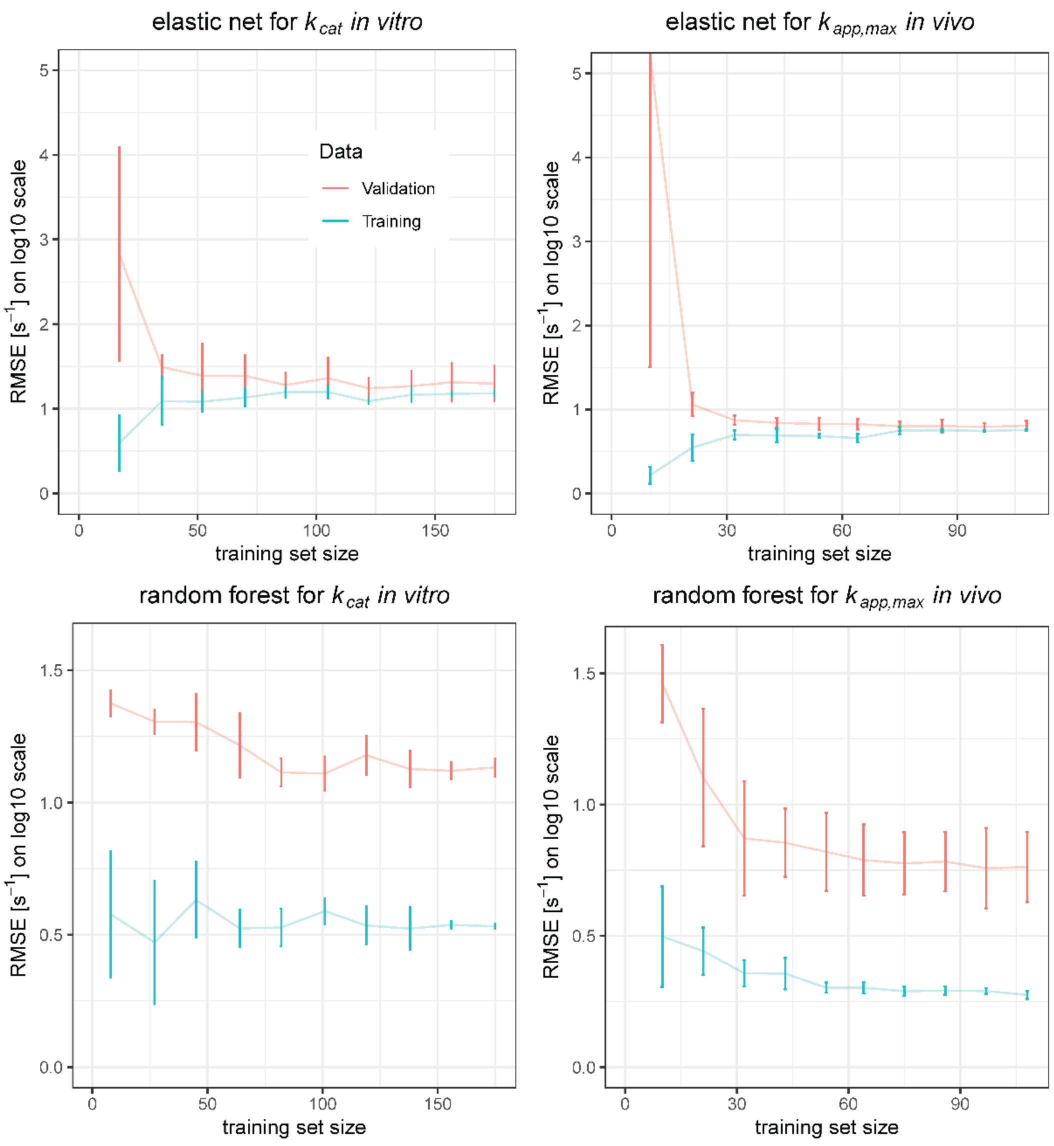

Supplementary Figure 4: Learning curves for elastic net and random forest model. 20% of the dataset of complete observations was set aside for the validation set on which the validation error is calculated. The model hyperparameters were set constant to the optimized value found in 5-fold cross-validation (as shown in Figure 2), and the training error was calculated using the model on the respective sub-sampled training set. The whole procedure was repeated three times, and the mean error across the three runs is shown along with the standard deviation as error bars. The slight decrease in RMSE with increasing training set size for random forest is due to the algorithm for decision tree training: the depth of the tree is lower for small data sets.

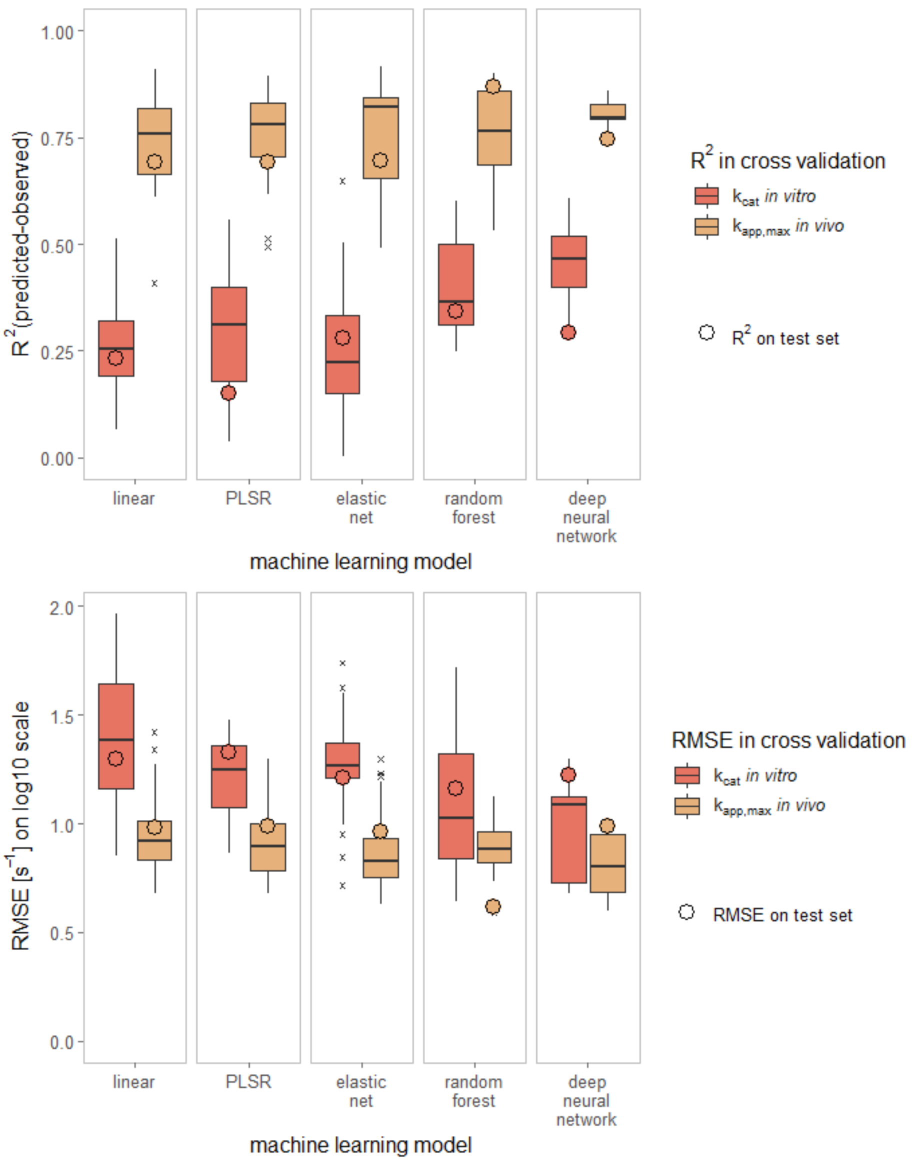

Supplementary Figure 5: Cross-validated machine learning model performances for $k_{app,max}$ and k_{cat} *in vitro* when using experimental flux data from metabolic flux analysis (MFA) as a feature. Related to Figure 2, where flux sampled from parsimonious FBA was used (see Methods). Here, we use MFA data under 8 growth conditions[1] to constrain FBA solutions and average flux across conditions to arrive at the flux feature (see Methods for details). Center lines show the median performance metric across 5 times repeated 5-fold cross-validation, except for the deep learning case, where a single round of 5-fold cross-validation is shown. Box limits represent the 1st and 3rd quartiles, whiskers extend to values that lie within the 1.5x interquartile range, and the remaining points are shown as outliers (marked x). Circles show the respective performance metric for a test set consisting of 20% of the available samples that were not used for hyperparameter optimization. This resulted in a training set of 113 observations of k_{cat} *in vitro* and 106 observations of $k_{app,max}$. For the test set, 29 and 27 observations were used for k_{cat} *in vitro* and $k_{app,max}$, respectively. See Methods for details on hyperparameter optimization.

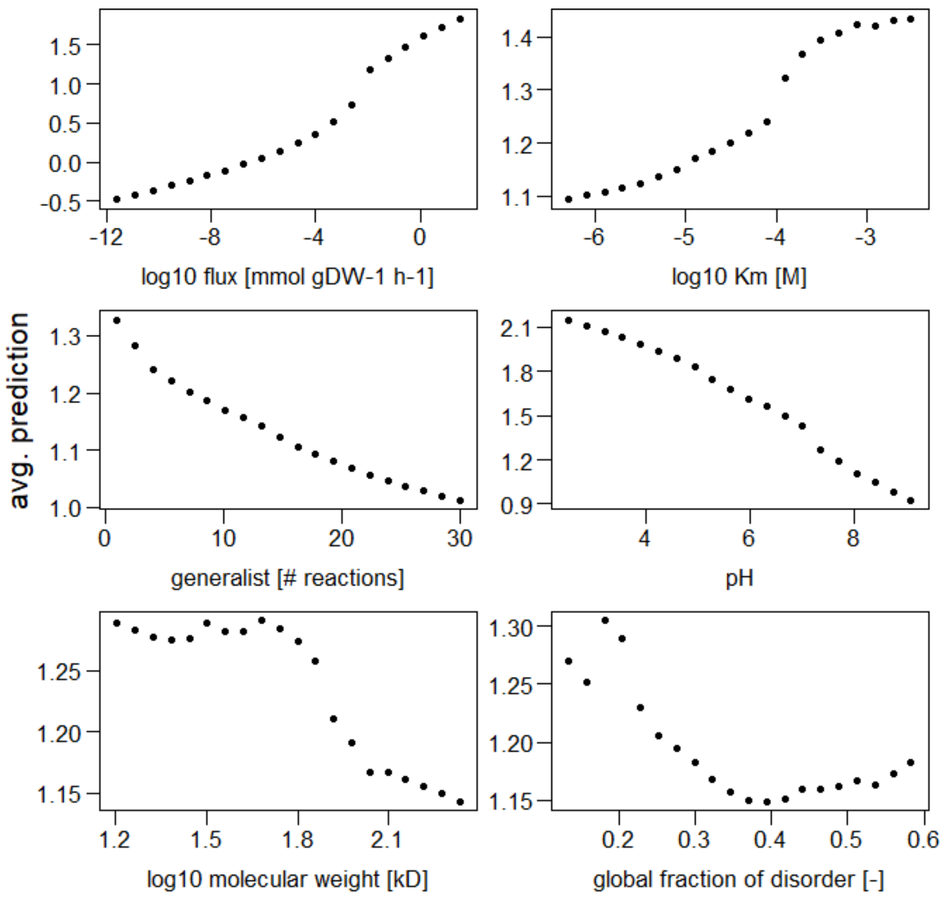

Supplementary Figure 6: Partial dependence plots for the k_{cat} *in vitro* ensemble model. The average output of the model in response to variation of the six most important statistically significant features are shown (see Figure 3).

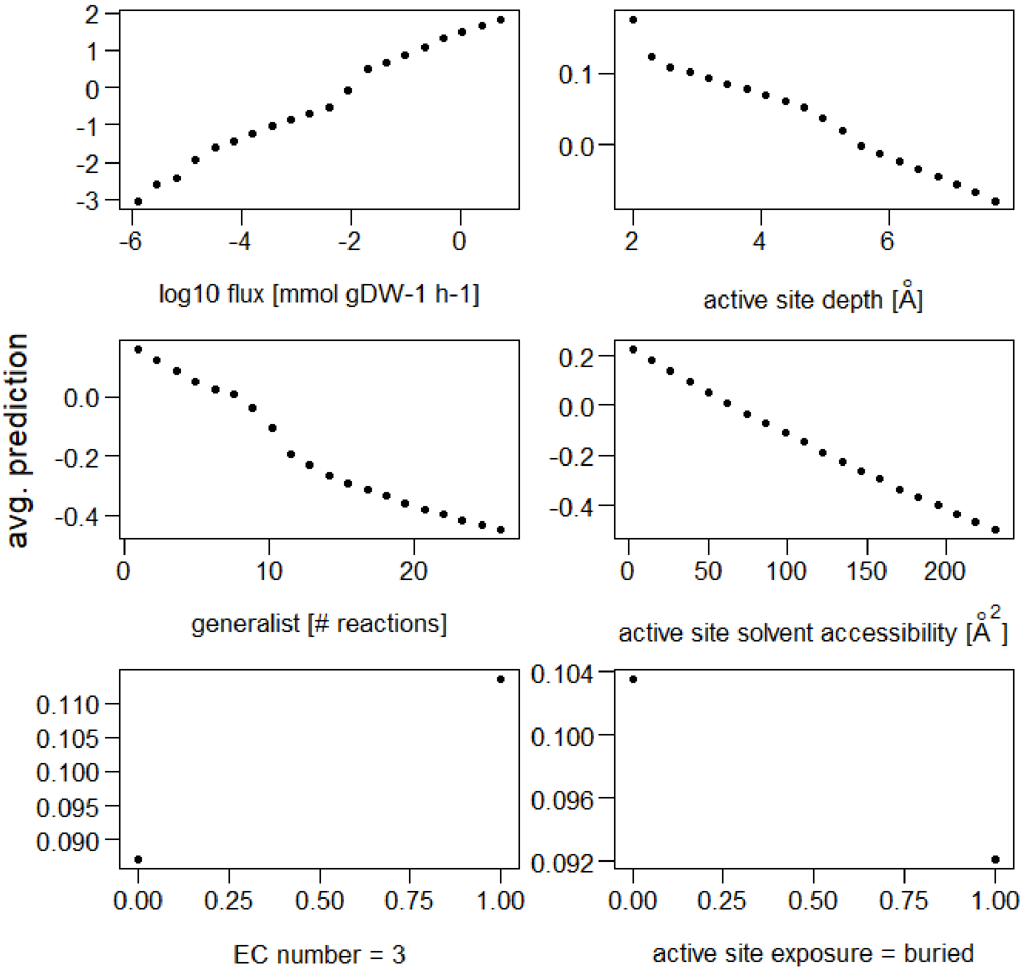

Supplementary Figure 7: Partial dependence plots for the $k_{app,max}$ ensemble model. The average output of the model in response to variation of the six most important statistically significant features are shown (see Figure 3).

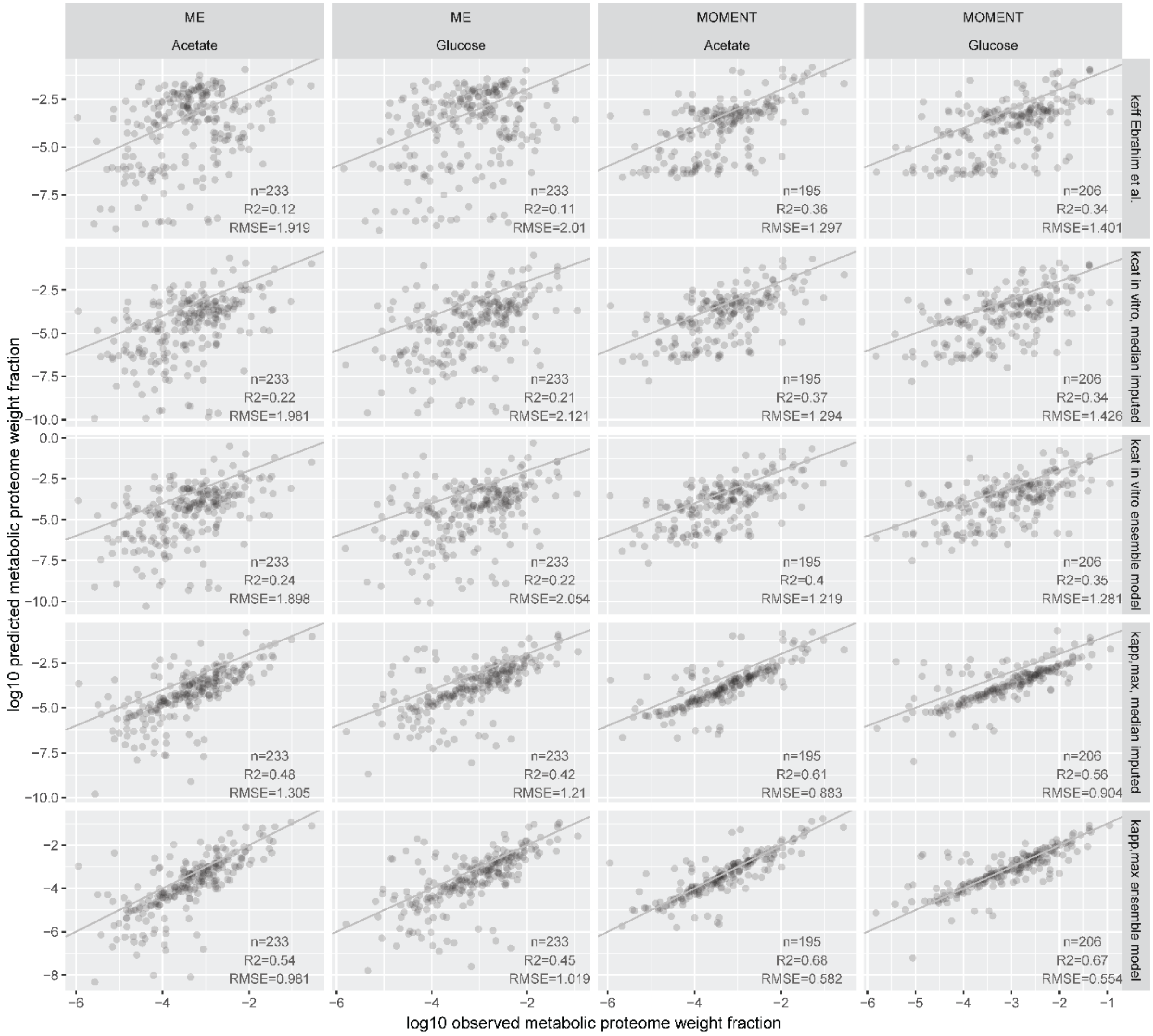

Supplementary Figure 8: Comparison of observed and predicted metabolic proteome weight fractions for growth on glucose and acetate. Related to Figure 4. Model predictions are compared to quantitative proteomics data in Schmidt et al.[2] for metabolic proteome fractions on log10 scale. Comparisons use proteins that are both found in proteomics data and are expressed in the model predictions. To allow comparison of different parameterization strategies, the intersection of the sets of comparable proteins is used in each condition-model combination resulting in the number of comparisons n. Grey lines are identity lines (slope=1, offset=0).

Supplementary Tables

Supplementary Table 1: Details on modelling features. Also see Methods.

**feature name**	**description**	**transformation**	**summarization to reaction level**	**class**
flux	average flux across random environments, predicted by parsimonious FBA (see Methods)	log10	-	numeric
active site depth	calculated residue depth for the carbon alpha atom (MSMS[3]), average over residues	-	average over participating genes	numeric
active site exposure	predicted exposed (e) or buried (-) residue (SCRATCH[4]), average over residues	-	majority over participating genes	categorical
active site hydrophobicity	the calculated hydrophobicity of the residue based the sequence, using a sliding window length of 7 residues and the Kyte-Doolittle scale for hydrophobicity[5], average over residues	-	average over participating genes	numeric
active site secondary structure	predicted secondary structure, 3 definitions (from the SCRATCH program[4]), majority over residues	-	majority over participating genes	categorical
global structural disorder	percent disordered residues in protein (from DSSP[6])	-	mean over components and isoforms	numeric
generalist	number of reactions that enzyme components are involved in	-	max over components and isoforms	numeric
EC number	first digit of EC number	-	-	categorical
number of active site residues	number of active site residues (from Catalytic Site Atlas[7])	-	average over participating genes	numeric
number of substrates	number of substrates involved in reaction	-	-	numeric
molecular weight	enzyme molecular weight	log10	Sum over components, average over isoforms	numeric
substrate concentration	Average substrate concentration across eight growth conditions[1]	log10	Average over individual substrate concentrations	numeric
product concentration	Average product concentration across eight growth conditions[1]	log10	Average over individual product concentrations	numeric
Thermodynamic efficiency	Average thermodynamic efficiency across eight growth conditions[1]	-	-	numeric
Km	Michaelis constant	log10	Average over all available substrate Kms	numeric
temperature	assay temperature (for *in vitro* data)	1/T	-	numeric
pH	assay pH (for *in vitro* data)	-	-	numeric

Supplementary Table 2: Statistical models and hyperparameters trained for turnover rate predictions.

model	implementation	hyperparameters	details	values in optimization
linear regression	R base::lm	-	-	-
PLSR	R pls	number of components	-	14 equidistant values between 1 and 14
elastic net	R elasticnet	L1 fraction	ratio of the L1 norm of the coefficient vector, relative to the norm at the full least squares solution	5 equidistant values between 0.3 and 0.8
		Lambda	weight on L2 regularization	10 equidistant values between 0 and 0.1
random forest	R randomForest	m tries	Number of variables randomly sampled as candidates at each split	10 equidistant values between 2 and 20
deep learning	R h2o	activation function		Random discrete search on {Rectifier, Tanh, Maxout, RectifierWithDropout, TanhWithDropout, MaxoutWithDropout}
		architecture	hidden layer sizes	Random discrete search on {(20,20),(50,50),(30,30,30),(25,25,25,25)}
		dropout ratio	hidden layer dropout ratio (if activation function allows for it)	Random discrete search on {0,0.05,0.1,0.2,0.5}
		L1 regularization		Random discrete search between 0 and 1e-4 in steps of 1e-6
		L2 regularization		Random discrete search between 0 and 1e-4 in steps of 1e-6
		learning rate		Random discrete search on 10 equidistant steps between 1e-4 and 1e-3
		Rho	adaptive learning rate time decay	Random discrete search on {0.9 , 0.99}
		epsilon	adaptive learning rate time smoothing factor	Random discrete search on {1e-09 , 1e-08 , 1e-07}

Supplementary References

1. Gerosa, L. *et al.* Pseudo-transition Analysis Identifies the Key Regulators of Dynamic Metabolic Adaptations from Steady-State Data. *Cell Syst* **1**, 270–282 (2015). doi:10.1016/j.cels.2015.09.008
2. Schmidt, A. *et al.* The quantitative and condition-dependent *Escherichia coli* proteome. *Nat. Biotechnol.* **34**, 104–110 (2016). doi:10.1038/nbt.3418
3. Sanner, M. F., Olson, A. J. & Spehner, J. C. Reduced surface: an efficient way to compute molecular surfaces. *Biopolymers* **38**, 305–320 (1996). doi:10.1002/(SICI)1097-0282(199603)38:3<305::AID-BIP4>3.0.CO;2-Y
4. Cheng, J., Randall, A. Z., Sweredoski, M. J. & Baldi, P. SCRATCH: a protein structure and structural feature prediction server. *Nucleic Acids Res.* **33**, W72–6 (2005). doi:10.1093/nar/gki396
5. Kyte, J. & Doolittle, R. F. A simple method for displaying the hydropathic character of a protein. *J. Mol. Biol.* **157**, 105–132 (1982).
6. Kabsch, W. & Sander, C. DSSP: definition of secondary structure of proteins given a set of 3D coordinates. *Biopolymers* **22**, 2577–2637 (1983).
7. Porter, C. T., Bartlett, G. J. & Thornton, J. M. The Catalytic Site Atlas: a resource of catalytic sites and residues identified in enzymes using structural data. *Nucleic Acids Res.* **32**, D129–33 (2004). doi:10.1093/nar/gkh028
