# Supplementary material for: Machine learning applied to enzyme turnover numbers reveals protein structural correlates and improves metabolic models
Source: Nat Commun. 2018 Dec 7;9:5252. doi: 10.1038/s41467-018-07652-6 (PMC6286351; doi:10.1038/s41467-018-07652-6)
Supplement: Supplementary file 3 — Description of Additional Supplementary Files [file 41467_2018_7652_MOESM3_ESM.docx]

**Description of Supplementary Information Files**

**File Name:** Supplementary Data 1

**Description:** E. coli K-12 MG1655 vectors of kcat in vitro and kapp,max imputed with median or ensemble models as used for Figure 4. Predictions for transporters and outer membrane proteins were out of scope for our models and are not included.
